# Wind, Waves, and Wing Loading: Morphological Specialization May Limit Range Expansion of Endangered Albatrosses

**DOI:** 10.1371/journal.pone.0004016

**Published:** 2008-12-24

**Authors:** Robert M. Suryan, David J. Anderson, Scott A. Shaffer, Daniel D. Roby, Yann Tremblay, Daniel P. Costa, Paul R. Sievert, Fumio Sato, Kiyoaki Ozaki, Gregory R. Balogh, Noboru Nakamura

**Affiliations:** 1 Oregon State University, Hatfield Marine Science Center, Newport, Oregon, United States of America; 2 Department of Biology, Wake Forest University, Winston-Salem, North Carolina, United States of America; 3 Department of Ecology and Evolutionary Biology, University of California Santa Cruz, Long Marine Laboratory, Santa Cruz, California, United States of America; 4 USGS-Oregon Cooperative Fish and Wildlife Research Unit, Department of Fisheries and Wildlife, Oregon State University, Corvallis, Oregon, United States of America; 5 USGS-Massachusetts Cooperative Fish and Wildlife Research Unit, Department of Natural Resources Conservation, University of Massachusetts at Amherst, Amherst, Massachusetts, United States of America; 6 Yamashina Institute for Ornithology, Abiko, Chiba, Japan; 7 U.S. Fish and Wildlife Service, Ecological Services, Anchorage, Alaska, United States of America; University of Sheffield, United Kingdom

## Abstract

Among the varied adaptations for avian flight, the morphological traits allowing large-bodied albatrosses to capitalize on wind and wave energy for efficient long-distance flight are unparalleled. Consequently, the biogeographic distribution of most albatrosses is limited to the windiest oceanic regions on earth; however, exceptions exist. Species breeding in the North and Central Pacific Ocean (*Phoebastria* spp.) inhabit regions of lower wind speed and wave height than southern hemisphere genera, and have large intrageneric variation in body size and aerodynamic performance. Here, we test the hypothesis that regional wind and wave regimes explain observed differences in *Phoebastria* albatross morphology and we compare their aerodynamic performance to representatives from the other three genera of this globally distributed avian family. In the North and Central Pacific, two species (short-tailed *P. albatrus* and waved *P. irrorata*) are markedly larger, yet have the smallest breeding ranges near highly productive coastal upwelling systems. Short-tailed albatrosses, however, have 60% higher wing loading (weight per area of lift) compared to waved albatrosses. Indeed, calculated aerodynamic performance of waved albatrosses, the only tropical albatross species, is more similar to those of their smaller congeners (black-footed *P. nigripes* and Laysan *P. immutabilis*), which have relatively low wing loading and much larger foraging ranges that include central oceanic gyres of relatively low productivity. Globally, the aerodynamic performance of short-tailed and waved albatrosses are most anomalous for their body sizes, yet consistent with wind regimes within their breeding season foraging ranges. Our results are the first to integrate global wind and wave patterns with albatross aerodynamics, thereby identifying morphological specialization that may explain limited breeding ranges of two endangered albatross species. These results are further relevant to understanding past and potentially predicting future distributional limits of albatrosses globally, particularly with respect to climate change effects on basin-scale and regional wind fields.

## Introduction

**Figure 1:**
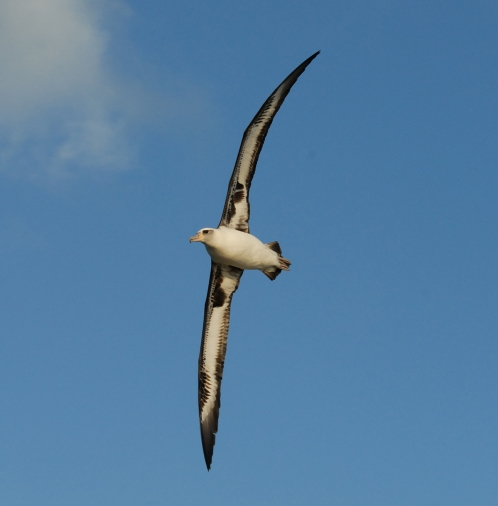
Wings locked in place, albatrosses expend little energy while dynamic soaring - repeated ascending and descending glide cycles through wind gradients near the ocean's surface - and avoiding flapping flight. Photo by D.P. Costa.

Albatrosses have captivated seafarers for centuries with their seemingly effortless flight over vast oceans, even in storm force winds [1], [2]. Indeed, modern tracking studies confirm that albatrosses are consummate travelers, routinely covering 1000 s of km from their breeding colonies in search of food for nestlings, as well as during post-breeding seasonal migrations [3], [4]. Success in exploiting resources in remote regions of the oceans is attributed to morphological adaptations, their ability to soar dynamically (Fig. 1), and their strategic use of wind systems - parasitizing wind and wave energy to minimize energetic costs of flight [5]–[8], and permitting some individuals to circumnavigate the Southern Ocean in just 46 days [9]. However, even albatrosses possess variability in body and wing morphology that have important implications for differential use of wind among species [10] and between sexes within a species [11], [12].

**Figure 2:**
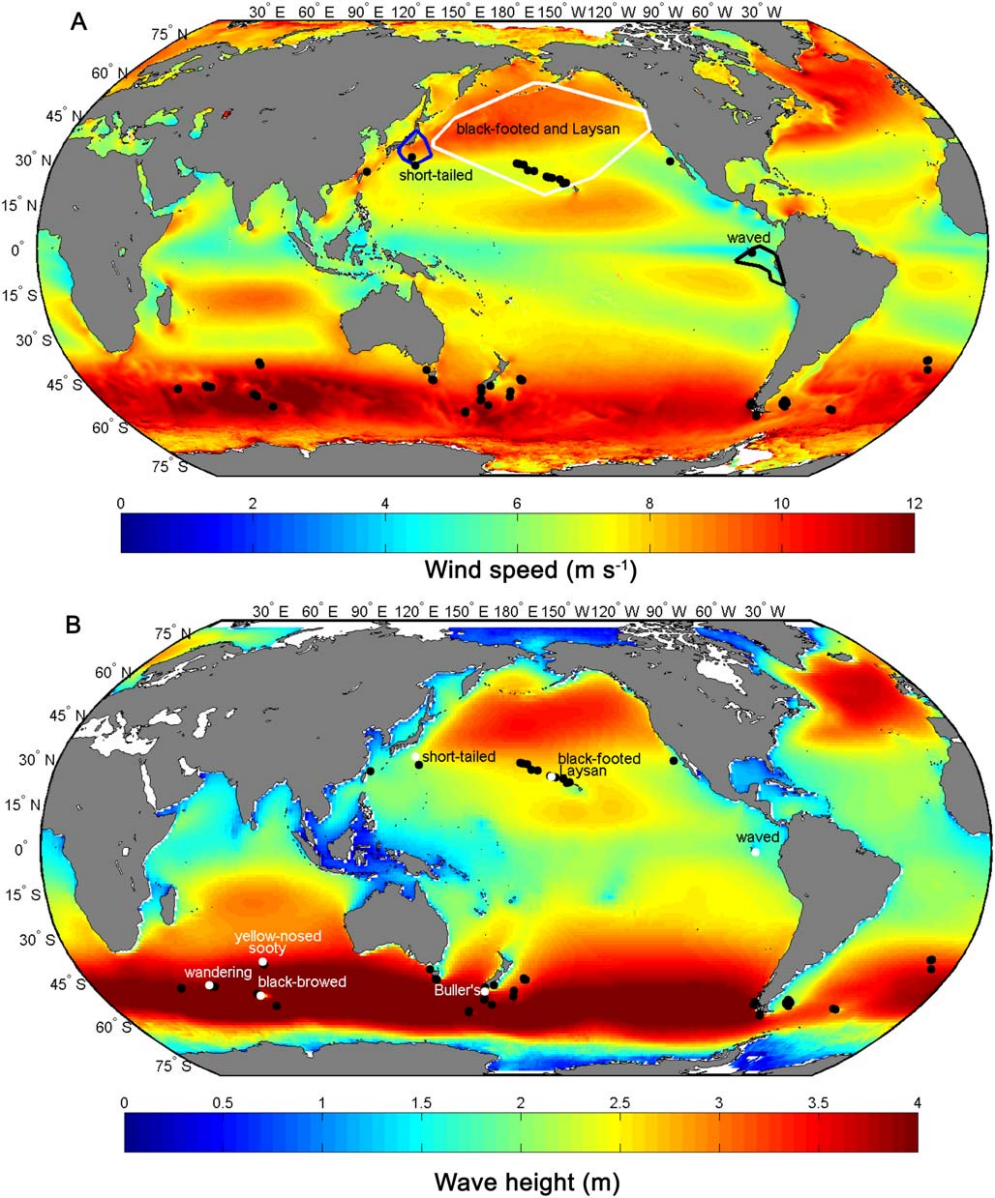
Global wind speed and wave height and the locations of albatross breeding colonies. (A) Eight-year (1999–2007) composite wind speed (m s^−1^, 0.25° resolution). Polygons encompass breeding season satellite-tracking locations of the four North and Central Pacific albatross species. Locations of global albatross colonies (≥20 pairs) are shown (black dots). (B) Eight-year (1999–2007) composite wave height (m, 1° latitude×1.25° longitude resolution) and locations of seven islands where albatross morphometric data were collected (white dots) with species listed.

Most albatross species (genera *Diomedea*, *Thalassarche*, *Phoebetria*) breed within a relatively narrow latitudinal band in the southern hemisphere where average wind speeds and wave heights are among the Earth's greatest (Fig. 2A,B). In contrast, four Pacific *Phoebastria* species span a broad latitudinal range including tropical to temperate regions of low to moderate average wind speeds and wave heights (Fig. 2A,B). Furthermore, although *Phoebastria* species are most phylogenetically similar to the largest albatrosses (*Diomedea* spp.) [13], they have a wide range in body size and vary markedly in species-specific biogeographic distribution. Medium-bodied short-tailed albatrosses breed only in the northwest Pacific [14] near (<1000 km) foraging sites within the productive Kuroshio and Oyashio coastal current systems [15]. Likewise, medium-bodied waved albatrosses breed almost exclusively in the equatorial Galápagos Islands and forage in the coastal Peruvian Upwelling (<1500 km away) [16], [17]. Conversely, the smaller-bodied black-footed and Laysan albatrosses primarily nest on islands in the central North Pacific [18] and traverse large expanses of open ocean (>3,000 km) during the breeding season to feed in the sub-Arctic transition zone and along continental shelf regions of the California and Alaska Currents [19], [20]. Herein, we examine whether body size and aerodynamic relationships of *Phoebastria* are consistent with other albatross genera and whether regional wind and wave regimes explain observed differences in albatross morphology

## Results and Discussion

**Figure 3:**
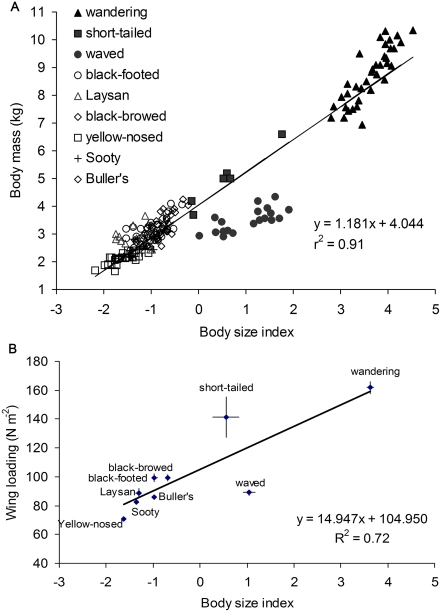
Albatross morphology and wing loading (weight per area of lift) of nine species representing all four genera. (A) Body mass *vs.* body size as indexed by the first principal component for measurements of culmen, tarsus, body girth, and wingspan, which collectively explained 91% of the variance in body measures. (B) Mean (±SE) wing loading *vs.* body size index for the same nine albatross species.

Morphologically, representatives from all four albatross genera show a very strong relationship between structural body size and mass (r^2^ = 0.91, F_1,194_ = 1902, *P*<0.001), although waved albatrosses, the only species with all negative residuals, present an obvious outlier in this relationship (Fig. 3A; r^2^ = 0.97 when excluding waved albatrosses). Aerodynamically, however, both medium-bodied short-tailed and waved albatrosses are outliers in a strong positive relationship between body size and wing loading (r^2^ = 0.72, F_1,7_ = 17.8, *P* = 0.004); short-tailed having anomalously high and waved anomalously low wing loading (Fig. 3B). In contrast, the smaller black-footed and Laysan albatrosses have body size-wing loading relationships similar to those of much larger bodied genera. Previous investigators suggested that differences in wing loading as small as 13% appeared sufficient to affect the distribution of albatrosses relative to wind speeds [11], [12]. Differences that we report among *Phoebastria* species are considerably larger, up to 60%, and, therefore, could conceivably permit exploitation of wind regimes by some, yet restrict others.

**Figure 4:**
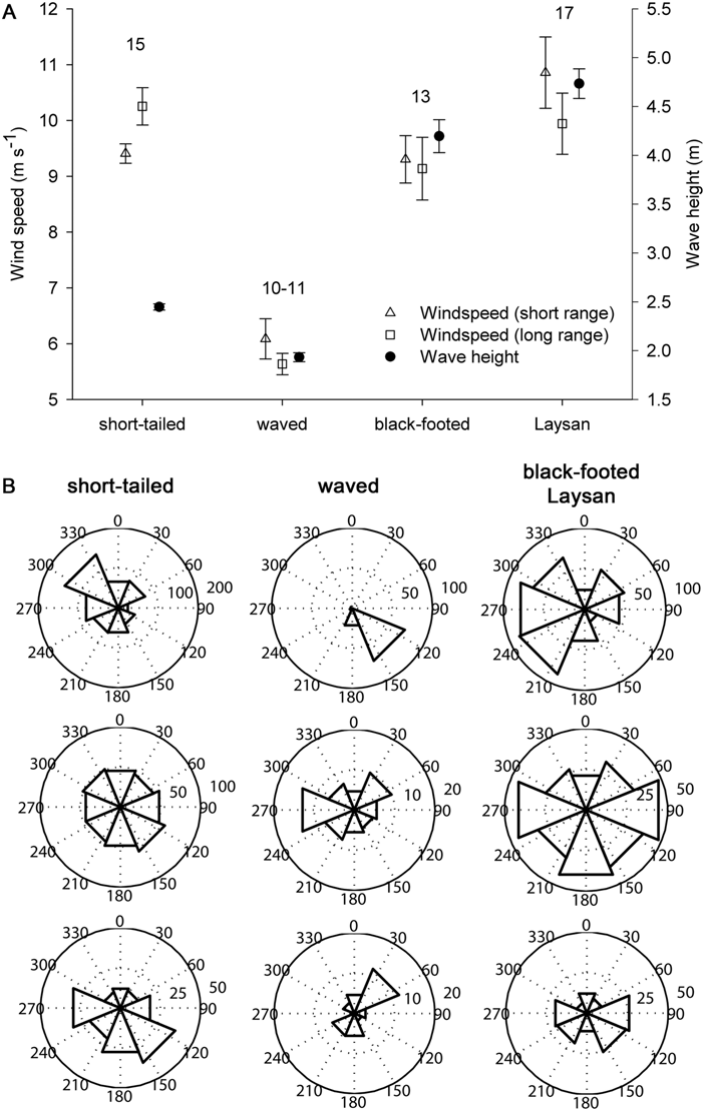
Wind velocities (speed and direction) and wave heights encountered by satellite tracked North and Central Pacific (*Phoebastria*) albatrosses. (A) Mean (±SE) wind speeds during short and long distance movements. Also shown are mean (±SE) wave heights encountered, regardless of distance traveled. Means are among individuals (*n* above symbols) for each species. (B) Polar histograms of true wind direction encountered (top row) and apparent wind direction (relative to net albatross trajectory) during short-distance (<100 km; middle row) and long distance (≥100 km; bottom row) movements. Values adjacent to concentric rings denote number of observations per bin (8 bins of 45° each).

Analyses of aerodynamic efficiencies and at-sea movements relative to wind and waves further exemplify variation among *Phoebastria* species. Glide polar calculations confirmed that short-tailed albatrosses had 15–25% greater minimum sink and best glide velocities, whereas waved albatrosses had lower values, comparable to black-footed and Laysan albatrosses (analysis of variance: F_3,67_ = 16.99 and 13.37, respectively, *P*<0.001; Table S1). Satellite-tracked short-tailed, black-footed, and Laysan albatrosses encountered similarly high wind speeds during the breeding season (9–11 m s^−1^), nearly twice those encountered by waved albatrosses (5–6 m s^−1^; mixed model ANOVA with bird as a random effect, *F*_3,55_ = 40.32, *P*<0.001; Fig. 4A). Short-tailed albatrosses, also having the highest wing loading, were the only species to travel long distances under windier conditions compared to their short-range movements (Fig. 4A). Only small-bodied black-footed and Laysan albatrosses encountered average wind speeds that exceeded their minimum sink velocities.

Through behavioral modifications, albatrosses can further enhance flight efficiency by optimizing their use of changing wind systems [21]. While at-sea, satellite-tracked waved albatrosses encountered consistent southeasterly trade winds, whereas wind direction was more variable for other species (Fig. 4B). During short distance movements (<100 km day^−1^), all species showed indiscriminate movement relative to the wind (Fig. 4B). During long-distance movements, however, all species except waved albatrosses supported the hypothesis of avoiding travel into headwinds (Fig 4B). The waved albatross' exception of flying frequently at 45° to the wind is best explained by the prevailing southeast trade winds that force birds to travel into a headwind between the Galapagos Islands and their feeding grounds off Ecuador and Perú [19] (Fig. 4B). Given this restriction, the low average wind velocity in this region may reduce the energetic costs of upwind flight [6], which is typically a pattern of “tacking” back and forth across wind to produce a net upwind movement trajectory [22], [23].

**Figure 5:**
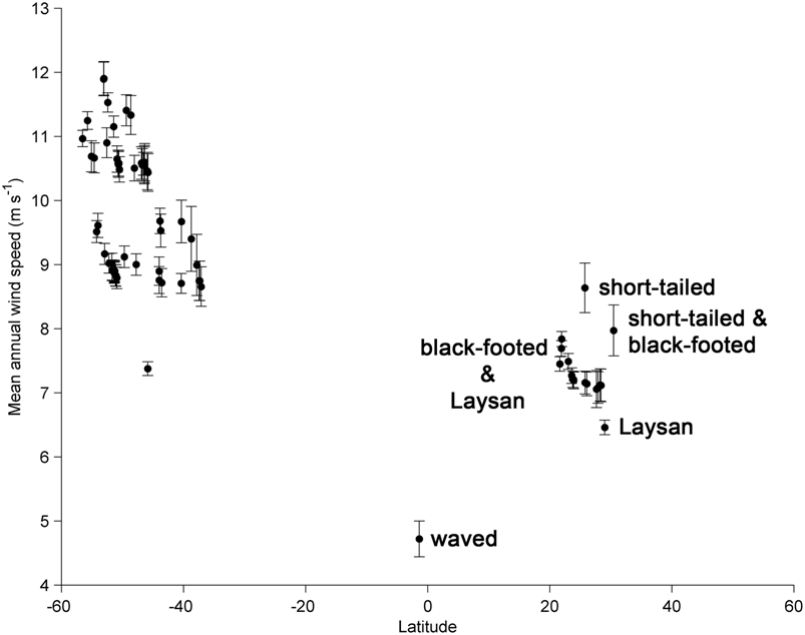
Mean (±SE) annual wind speed within 100 km radius of global albatross colonies *vs.* latitude. Wind data are from an eight year (1999–2007) time series and summarized for albatross colonies containing ≥20 breeding pairs.

The breeding range of much heavier short-tailed albatrosses has been confined in recent history to the lower mid-latitudes of the western North Pacific [14]. For a central-place forager with such high wing loading, the western North Pacific region is particularly advantageous because: 1) their primary foraging grounds (waters over continental shelf break-slope habitats, upwelling plumes of islands and passes, and submarine canyons [15], [24] are nearby, relative to other albatross colonies in the Central and North Pacific and 2) frequent cyclonic and anti-cyclonic weather systems from the east Asian continent move west to east through the region, allowing favorable traveling winds by simply timing northward and southward movements to/from foraging grounds. These variable wind patterns (e.g., angular dispersion [s] = 73.1 within 100 km of Torishima, Japan) contrast with the relatively consistent trade winds at albatross colonies in other regions of the North and Central Pacific (e.g., s = 13.3 near Tern Island, Hawaii and s = 28.6 near Isla Española, Galapagos). Both of the aforementioned advantages were likely a benefit to short-tailed albatrosses breeding at historical sites in the western North Pacific, which have lower average wind speeds than current breeding sites. Currently short-tailed albatrosses breed on islands where regional wind speeds are higher relative to other albatross breeding colonies in the North Pacific, but still less, on average, than nearly all southern hemisphere colonies (Fig. 5).

Although considered a medium-sized albatross, the rather bulky short-tailed albatross has minimum sink and best glide velocities approaching those (only 25% less) of the much larger wandering albatross (*Diomedea exulans*), which is essentially restricted to only the windiest regions of the southern hemisphere, at least during the breeding season [6], [11]. During the non-breeding season, however, short-tailed albatrosses ventured into regions with low wind speeds (median = 6.26±0.54 m s^−1^) and wave heights (mean = 2.17±0.12 m) near continental margins, apparently seeking their preferred foraging areas, despite windier regions offshore. This behavior could entail greater flight costs if birds are forced to use flapping flight to augment travel under low wind conditions [6]. Higher flight costs, however, could be offset by reduced travel times and non-central-place foraging in highly productive regions compared to acquiring equivalent forage over a larger area, but under windier conditions. Indeed, in the Southern Ocean, it is primarily within the productive boundary current systems of South America and Africa that albatrosses regularly venture into low latitudes north of 30° S [25], despite encountering lower average wind speeds wave heights in these regions compared to higher latitudes (Fig. 2). Like the short-tailed albatross, the waved albatross forages in a highly productive upwelling system; however, unlike short-tailed, waved albatrosses encounter low wind speeds year-round. Fittingly, the waved albatross had aerodynamic performances more similar to smaller albatrosses, requiring less wind to stay aloft than the short-tailed, despite being similar in body size. Hence, the waved albatross appears adapted for a low wind speed and wave height environment and, indeed, is the only albatross to breed in a tropical ocean. This disparity in aerodynamic performance among albatrosses appears to have important implications for flight energetics with respect to regional wind patterns and may help explain breeding range limitations of these two endangered *Phoebastria* albatrosses.

These patterns in morphology affecting aerodynamic performance and distribution could result from recent adaptation at the species level, but they might also reflect the pursuit of similar environments by morphologically similar close relatives, without recent adaptation. Indeed black-footed and Laysan albatrosses are more closely related to each other than to other *Phoebastria* species [13], they are the most similar morphologically, and their breeding distributions have the greatest overlap. In contrast, Short-tailed and waved albatrosses also are sister taxa [13], yet they differ dramatically in aerodynamic performance and occupy the most oceanographically divergent regions, consistent with effects of both local adaptation and phylogeny confining the larger *Phoebastria* species to specific oceanographic regions.

Breeding ranges of *Phoebastria* albatrosses are further constrained by confinement within a single ocean basin, with the aerodynamic performance of short-tailed albatrosses likely preventing crossing through areas of low wind speed and low productivity (e.g. central Pacific) and the aerodynamic performance of waved albatrosses potentially impairing efficient use of windy areas. This is in strict contrast to high latitude southern hemisphere albatrosses, which have the potential to transit all southern oceans and colonize new islands unimpeded by land masses (Fig. 2). Several albatross species, including short-tailed, historically nested in the North Atlantic Ocean, prior to the inter-glacial period of the middle Pleistocene [26]. Sea level rise during this period appeared to have extirpated the last albatross colony in the Atlantic (short-tailed in this case), at which point the Panamanian seaway was closed leaving the North Atlantic isolated from recolonization by *Phoebastria* species. Currently, many albatross colonies in the central North Pacific are on low lying atolls and similarly subject to extirpation by projected sea level rise [27], potentially further restricting breeding ranges of *Phoebastria* in general. Hence, restoration of remote, predator free, and higher elevation island habitats may be particularly important for the long-term conservation of *Phoebastria* species; especially islands within productive continental margins for the two endangered species that show specialization for breeding in these regions.

## Materials and Methods

Albatross morphology (*n* = 221 individuals) was measured for nine species (representing all four genera of the 22 extant albatross species) from seven islands (plus one at-sea location for short-tailed albatrosses) in the North and South Pacific and Indian Oceans (Fig 2B). Albatross wing shape, body size, and flight performance characteristics (Table S1) were collected and analyzed using standard methods [10], [11], [28], [29]. Satellite transmitters (*n* = 74) were attached to Pacific *Phoebastria* albatrosses. Birds tracked were not always the same as those measured and only one individual per pair was tagged or measured. We captured short-tailed albatrosses at the breeding colony on Torishima, Japan (Fig. 1B), and at-sea near Seguam Pass (52° 25.8′ N, 172° 46.4′ W), Alaska. Short-tailed albatrosses were tracked during the winter, chick-rearing period (∼30 days of age to fledging) and summer post-breeding migrations (Table S2). Data for breeding (central-place foraging) and post-breeding (migratory) short-tailed albatrosses were analyzed separately. Waved albatrosses were measured and tracked from their breeding colony on Isla Española, Galapagos Islands, Ecuador, and black-footed and Laysan albatrosses from Tern Island, French Frigate Shoals, Northwestern Hawaiian Archipelago (Fig. 1B). Waved, black-footed, and Laysan albatrosses were tracked during the winter incubation and chick-brooding periods (chicks≤18 days; Table S2). Measurements of southern hemisphere species were obtained from wandering (*Diomedea exulans*; *n* = 56, Iles Crozet), black-browed (*Thalassarche melanophrys*, *n* = 20, Iles Kerguelen), Indian yellow-nosed (*T. carteri*, *n* = 25, Iles Amsterdam), Buller's, (*T. bulleri*, *n* = 30, Snares Islands) and sooty albatrosses (*Phoebetria fusca*, *n* = 14, Iles Amsterdam). It was not feasible to adequately sample various age classes and both sexes of all species in this study. Therefore, our hypotheses focus on inter-specific differences rather than age and gender effects.

### Satellite tracking

We used conventional Argos satellite tracking and data filtering techniques previously used for these species [15], [19], [20] (Table S2). Transmitters operated on varying duty cycles but were standardized for analyses by sub-sampling to a maximum of 6–8 hr on per day, providing from 2.3 (range 1.3–3.6) locations per individual per day in low-latitudes to 5.1 (range 2.6–8.3) in higher latitudes (*n* = 13,624 locations for all four species combined; Table S2).

### Wind and wave data

We obtained global QuikSCAT wind data for 10 m above the ocean surface (a height consistent with observations of albatrosses soaring [10]; http://www.remss.com; sponsored by the NASA Ocean Vector Winds Science Team). For analyses of geographic patterns of wind regimes near colonies, we used monthly composites. For analyses of wind vectors along albatross tracks, we averaged twice daily satellite passes to create daily composites.

We obtained data on significant wave heights from the U.S. National Oceanic and Atmospheric Administration (NOAA), National Center for Environmental Prediction (http://polar.ncep.noaa.gov/waves/Welcome.html). Significant wave height represents the average of the highest 33% of all individual waves. We used data output from the NOAA Wavewatch III model. Model inputs include wind velocity, sea surface temperature, ice conditions, and land boundaries. We calculated 24-hr averages for determining wave heights encountered by albatrosses during daily movement trajectories.

### Analysis of albatross tracks with wind and wave data

Albatross locations for a given day were used if there were two or more locations separated by at least one hour. We extracted wind speed and direction within a 15-km buffer along the albatross fight path to represent local wind conditions encountered for a given day. Because the resolution of wave data was approximately five times coarser than the wind data, we used a 75-km buffer to represent local wave conditions. We calculated mean wind velocity and wave height among albatross locations, and net distance traveled and net direction of movement between the first and last location of each day. We differentiated long distance (net trajectories of ≥100 km - approximately the upper quartile in distances traveled during a 6–8-hr transmitter “on cycle”) from short distance movements (<100 km during a 6–8-hr transmitter “on cycle”) because albatross behavior with respect to wind and wave fields likely differ between transitory (long) vs. local (short distance) movements.

### Statistical analyses

We used the individual bird as a sampling unit (*n* = number of birds) to standardize for varying numbers of locations, tracking days, and foraging trips among birds. We calculated mean angular deviation to compare variability in wind direction, where values range from 0° (no dispersion) to 81.03° (maximum dispersion). Results of statistical tests were considered significant if *P*<0.05. Multi-sample comparisons were conducted using standard or mixed effects ANOVA with a Tukey post-hoc test. If necessary, data were log_10_-transformed to meet conditions of normality or a Kruskal-Wallis non-parametric test was used.

## Supporting Information

Table S1 Mean (SE, *n*) body morphometrics and aerodynamic calculations for *Phoebastria* albatrosses (short-tailed, black-footed, and Laysan) in the North Pacific and waved in the Eastern Equatorial Pacific. Similar superscripted letters denote means that are not significantly different (*P*≥0.05 from multiple comparison tests). Maximum body frontal area (*S_b_*) was calculated from maximum body circumference (*C*) using the formula *S_b_* = *C^2^*/4π. Root chord (width at the most proximal end of wing) measured from each trace was multiplied by shoulder width to determine inter-wing area. Total wing area (*S*) is 2*area of trace+inter-wing area. Wing traces were converted to area using a mass-to-area linear regression determined by weighing sheets of paper of known area [11], [28]. Wing loading (*W*), a measure of force per unit area was calculated as (mass*gravity)/*S* and expressed as Newtons (N) m^−2^ (assuming gravity = 9.81 m s^−2^), mean wing chord (*c*) as *S*/wing span (*b*), and aspect ratio (*A*), a measure of aerodynamic efficiency, as *b/c*.
(0.06 MB DOC)

Table S2 Summary of satellite tracking data for North and Central Pacific albatrosses during 2001 to 2007. Ranges presented for number of tracking days and filtered locations represent minimum and maximum values for individuals from a given species, with the total in parentheses.
(0.03 MB DOC)
