# Supplementary material for: Wind, Waves, and Wing Loading: Morphological Specialization May Limit Range Expansion of Endangered Albatrosses
Source: PLoS One. 2008 Dec 24;3(12):e4016. doi: 10.1371/journal.pone.0004016 (PMC2602987; doi:10.1371/journal.pone.0004016)
Supplement: Table S1 — Mean (SE, n) body morphometrics and aerodynamic calculations for Phoebastria albatrosses (short-tailed, black-footed, and Laysan) in the North Pacific and waved in the Eastern Equatorial Pacific. Similar superscripted letters denote means that are not significantly different (P≥0.05 from multiple comparison tests). Maximum body frontal area (Sb) was calculated from maximum body circumference (C) using the formula Sb = C2/4π. Root chord (width at the most proximal end of wing) measured from each trace was multiplied by shoulder width to determine inter-wing area. Total wing area (S) is 2*area of trace+inter-wing area. Wing traces were converted to area using a mass-to-area linear regression determined by weighing sheets of paper of known area [11], [28]. Wing loading (W), a measure of force per unit area was calculated as (mass*gravity)/S and expressed as Newtons (N) m−2 (assuming gravity = 9.81 m s−2), mean wing chord (c) as S/wing span (b), and aspect ratio (A), a measure of aerodynamic efficiency, as b/c. (0.06 MB DOC) [file pone.0004016.s001.doc]

Table S1. Mean (SE*, n*) body morphometrics and aerodynamic calculations for *Phoebastria* albatrosses (short-tailed, black-footed, and Laysan) in the North Pacific and waved in the Eastern Equatorial Pacific. Similar superscripted letters denote means that are not significantly different (*P* > 0.05 from multiple comparison tests). Maximum body frontal area (*S_b_*) was calculated from maximum body circumference (*C*) using the formula *S_b_* = *C*^2^/4π. Root chord (width at the most proximal end of wing) measured from each trace was multiplied by shoulder width to determine inter-wing area. Total wing area (*S*) is 2*area of trace + inter-wing area. Wing traces were converted to area using a mass-to-area linear regression determined by weighing sheets of paper of known area [11,28]. Wing loading (*W*), a measure of force per unit area was calculated as (mass*gravity)/*S* and expressed as Newtons (N) m^-2^ (assuming gravity = 9.81 m s^-2^), mean wing chord (*c*) as *S*/wing span (*b*), and aspect ratio (*A*), a measure of aerodynamic efficiency, as *b*/*c*.

|  | short-tailed | waved | black-footed | Laysan | | *F* | *P* |
| --- | --- | --- | --- | --- | --- | --- | --- |
| Metrics for Glide Performance Calculations | | | | | | | |
| Mass (kg) | 4.68^a^  (0.13, 25) | 3.51^b^  (0.95, 19) | 3.17^b^  (0.76, 29) | 2.77^c^  (0.89, 18) | | 69.2 | < 0.001 |
| Girth (cm) | 60.3^a^  (1.7, 6) | 54.9^b^  (0.6, 19) | 49.9^c^  (0.6, 29) | 47.8^c^  (0.6, 23) | | 39.4 | < 0.001 |
| Body Frontal Area (cm^2^) | 291^a^  (17, 6) | 241^b^  (6, 19) | 199^c^  (5, 29) | 183^c^  (5, 23) | | 37.2^*^ | < 0.001 |
| Shoulder width (cm) | 19.2  (5.4, 6) | 17.5  (2.0, 19) | 15.9  (1.8, 29) | 15.2  (1.9, 23) | | -^**^ | - |
| Wing span (cm) | 228^a^  (2, 10) | 238^b^  (2, 19) | 221^c^  (2, 29) | 209^d^  (1, 23) | 59.1 | | < 0.001 |
| Wing area (cm^2^) | 3406^a^  (91, 5) | 3858^b^  (60, 19) | 3170^a,c^  (39, 29) | 3065^c^  (33, 23) | 57.4 | | < 0.001 |
| Wing chord (cm) | 14.8^a^  (0.4, 5) | 16.2^b^  (0.2, 19) | 14.4^a^  (0.1, 29) | 14.6^a^  (0.1, 23) | 32.7 | | < 0.001 |
| Aspect ratio | 15.6^a^  (0.5, 5) | 14.7^b^  (0.2, 19) | 15.4^a^  (0.1, 29) | 14.3^b^  (0.1, 23) | 13.0^*^ | | < 0.001 |
| Wing loading (N m^-2^) | 141.2^a^  (13.9, 5) | 89.2^b,c^  (2.0, 19) | 99.3^b^  (2.6, 28) | 88.7^c^  (3.3,18) | 16.8^*^ | | < 0.001 |
| Glide Performance^***^ |  |  |  |  |  | |  |
| Minimum sink velocity (m s^-1^) | 11.22 ^a^  (0.51, 5) | 8.95 ^b,c^  (0.10, 19) | 9.44^b^  (0.12, 28) | 8.93^c^  (0.17, 18) | 16.99^*^ | | < 0.001 |
| Minimum sink (m s^-1^) | 0.60^a^  (0.03, 5) | 0.49^b^  (0.004, 19) | 0.50^b^  (0.01, 28) | 0.49^b^  (0.01, 18) | 12.51 | | < 0.001 |
| Best glide velocity  (m s^-1^) | 14.70^a^  (0.55, 5) | 12.29^b^  (0.10, 19) | 12.77^b^  (0.14, 28) | 12.43^b^  (0.23, 18) | 13.37 | | < 0.001 |
| Best glide ratio | 20.80^a^  (0.25, 5) | 21.04^a^  (0.13, 19) | 21.55^b^  (0.12, 28) | 20.95^a^  (0.10, 18) | 6.07 | | 0.001 |

^*^Log transformed

^**^Shoulder width was approximated by calculating body diameter from girth measurements

^***^Calculated using computer program Flight v1.15 (29). For each bird, we input our measures of body mass, wing span, wing area, aspect ratio, air density at sea level (1.23 kg m^-3^), and body frontal area. Program default values were used for the remainder of inputs required to calculate glide polars. We excluded birds with incomplete sets of measurements.
