# Supplementary material for: Wind, Waves, and Wing Loading: Morphological Specialization May Limit Range Expansion of Endangered Albatrosses
Source: PLoS One. 2008 Dec 24;3(12):e4016. doi: 10.1371/journal.pone.0004016 (PMC2602987; doi:10.1371/journal.pone.0004016)
Supplement: Table S2 — Summary of satellite tracking data for North and Central Pacific albatrosses during 2001 to 2007. Ranges presented for number of tracking days and filtered locations represent minimum and maximum values for individuals from a given species, with the total in parentheses. (0.03 MB DOC) [file pone.0004016.s002.doc]

Table S2. Summary of satellite tracking data for North and Central Pacific albatrosses during 2001 to 2007. Ranges presented for number of tracking days and filtered locations represent minimum and maximum values for individuals from a given species, with the total in parentheses.

| Species | Individuals | Tracking  Days | Filtered Locations | Months Tracked |
| --- | --- | --- | --- | --- |
| short-tailed  breeding | 15 | 35 - 96  (1012) | 75 - 467  (3831) | Feb - May 2006  Feb – May 2007 |
| short-tailed  post-breeding | 14 | 51 - 125  (1262) | 131 - 808  (6709) | May - Sep 2002  May - Nov 2003 |
| waved | 14 | 7 – 38  (288) | 11 – 105  (717) | May – Jul 2001  May – Jul 2002 |
| black-footed | 14 | 6 – 21  (206) | 21 – 99  (890) | Dec – Feb 2002/03  Nov – Jan 2003/04 |
| Laysan | 17 | 4 - 32  (336) | 17 - 143  (1477) | Dec – Feb 2002/03  Nov – Jan 2003/04 |
